# Age-related neuropathies and tubulin acetylation

**DOI:** 10.18632/aging.101432

**Published:** 2018-04-28

**Authors:** Jaime Fernández-Barrera, Isabel Correas, Miguel A. Alonso

**Affiliations:** 1 Department of Cell Biology and Immunology, Centro de Biología Molecular Severo Ochoa, Consejo Superior de Investigaciones Científicas and Universidad Autónoma de Madrid, Madrid, Spain; 2 Department of Molecular Biology, Universidad Autónoma de Madrid, Madrid, Spain

**Keywords:** Alzheimer, Huntington and Parkinson diseases, tubulin acetylation, formins, INF2

Microtubules (MTs) are highly dynamic cytoskeletal polymers that are involved in a wide range of cellular processes such as cell polarity, morphogenesis and vesicular transport. The bulk of cytoskeletal MTs last only a few minutes but there is an MT subset with a much longer duration. This long-lived subset accumulates a number of covalent posttranslational modifications such as acetylation. The acetylation in the Lys^40^ residue of α-tubulin, which occurs in the MT lumen, weakens lateral inter-protofilament interactions, endowing MTs with greater flexibility and protecting them from mechanical stress. MT acetylation regulates intracellular vesicular transport by modulating the recruitment of kinesin-1 MT motors. Defects in MT acetylation are associated with age-related degenerative neuropathies such as Alzheimer, Huntington and Parkinson diseases, amongst others [1]. Consistent with a possible role for acetylated MT in vesicular transport, mouse models of Alzheimer disease show axonal defects consisting of abnormal amounts of MT-associated organelles and vesicles, similar to the case in the early stages of Alzheimer disease in humans [2]. As further evidence of the existence of a collapse in MT-mediated vesicular trafficking, reduction of the dosage of kinesin-1 enhances the frequency of axonal defects and increases amyloid-β peptide levels and amyloid deposition in these mice [3]. Leucine-rich repeat kinase 2 (LRRK2) mutations are the most common genetic cause of Parkinson disease. LRRK2 containing pathogenic mutations preferentially associates with deacetylated MTs, inhibits axonal transport in primary neurons and in a *Drosophila* model of this disease [4]. Neuropathogenic forms of huntingtin impair axonal transport *in vivo* and *in vitro*. However, no clear link has been established between huntingtin and the defects in MT acetylation observed in brains of patients with Hungtinton disease [5]. Therefore, although the exact role of tubulin acetylation in these diseases is still unclear, the hypothesis has emerged that the defect in tubulin acetylation is associated with dysfunctional MT-mediated axonal transport.

**Figure 1:**
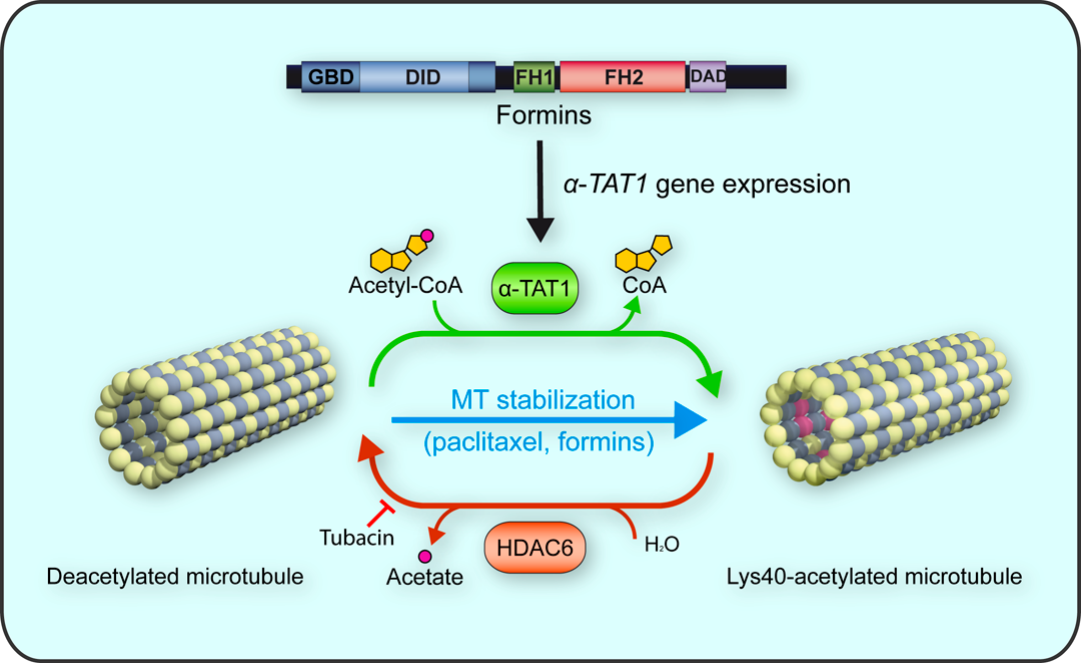
**Regulation of tubulin acetylation.** α-TAT1 and HDAC6 control the acetylation and deacetylation of Lys^40^ of α-tubulin, respectively. Paclitaxel treatment increases α-tubulin acetylation by augmenting MT stability, whereas HDAC6 inhibitors (e.g., tubacin) do so by reducing α-tubulin deacetylation. INF2 and other formins affect tubulin acetylation by regulating MT stability and by controlling the levels of α-TAT1 via expression of the *α-TAT1* gene.

In mammals, the levels of acetylated tubulin are regulated by reactions of acetylation and deacetylation, which are catalyzed by α-tubulin acetyltransferase 1 (α-TAT1) and histone deacetylase 6 (HDAC6), respectively [1] (Figure 1). Formins, which are a family of proteins whose primary function is to nucleate and polymerize monomeric globular actin (G-actin) into linear actin filaments, bind MTs and regulate MT stability. MT stabilization results from the capping of the MT ends by a large protein machinery complex that includes different formins, and MT plus-end tracking and scaffolding proteins, in a process promoted by the GTPase Rho and inhibited by glycogen synthase kinase 3β. Since this complex increases MT longevity, it contributes to the accumulation of acetylated Lys40 α-tubulin residues. Our recent work indicates that inverted formin 2 (INF2), which is a formin mutated in autosomal-dominant focal segmental glomerulosclerosis, with or without associated Charcot-Marie-Tooth neuropathy, modulates tubulin acetylation also by employing an MT stabilization-independent mechanism [6]. Silencing of INF2, but not of the diaphanous-related formin 1, dramatically impairs tubulin acetylation in such a way that neither pharmacological stabilization of MTs with paclitaxel (taxol) nor inhibition of the deacetylase HDAC6 with tubacin restores MT acetylation. The observations that α-TAT1 mRNA was nearly depleted in INF2-silenced cells and that exogenous expression of α-TAT1 restored MT acetylation indicate that the absence of α-TAT1 is the only cause of the lack of tubulin acetylation in INF2-silenced cells. Further work revealed that INF2 controls the expression of the α*-TAT1* gene via the myocardin-related transcription factor-serum response factor (MRTF-SRF) transcriptional complex by regulating the levels of G-actin. In the absence of INF2, G-actin levels increase, and G-actin binds to the MRTF coactivator, causing MRTF retention in the cytoplasm. Subsequently, the association of MRTF with SRF in the nucleus is impeded and the transcription of the *α-TAT1* gene is blocked. Therefore, INF2 modulates MT acetylation through two different mechanisms, one involving regulation of MT stability and the other the control of α-TAT1 levels.

Proteins that modulate the levels of MT acetylation are potential pharmacological targets for therapeutic strategies aimed at correcting the defects in tubulin acetylation found in age-related neurodegenerative diseases (Figure 1). For instance, paclitaxel increases tubulin acetylation and reverses dopaminergic neuron death in cultures of rat neurons treated with the Parkinson disease environmental toxin rotenone [7]. Inhibition of HDAC6 compensates for the intracellular protein transport deficit in striated precursor cells from knock-in mice that express neuropathogenic huntingtin [5]. HDAC6 inhibition reduces the level of protein tau and improves memory and, consistently, the reduced expression of HDAC6 ameliorates cognitive defects in mouse models of Alzheimer disease [2,3]. Since α-TAT1 identification as the α-tubulin acetylating enzyme is relatively recent compared with that of the HDAC6 deacetylase, its potential use as a therapeutic target has not yet been exploited. Given their dual role in MT acetylation by which they act on MT stability on one hand, and on the α-TAT1 levels on the other, formins, in particular INF2, have the potential to be novel therapeutic target compounds that can correct the alterations in tubulin acetylation that arise as part of age-related neurodegenerative diseases, or of other disorders featuring altered levels of acetylated tubulin.
